# Impact of Superselective Renal Artery Embolization on Renal Function and Blood Pressure

**DOI:** 10.5334/jbsr.2223

**Published:** 2020-11-06

**Authors:** Hyoung Nam Lee, Seung Boo Yang, Dong Erk Goo, Yong Jae Kim, Woong Hee Lee, Dongho Hyun, Nam Hun Heo

**Affiliations:** 1 Department of Radiology, Soonchunhyang University College of Medicine, Cheonan Hospital, Cheonan-si, KR; 2 Department of Radiology, Soonchunhyang University College of Medicine, Gumi Hospital, Gumi, KR; 3 Department of Radiology, Soonchunhyang University College of Medicine, Seoul Hospital, Seoul, KR; 4 Department of Radiology, Samsung Medical Center, Sungkyunkwan University School of Medicine, Seoul, KR; 5 Clinical Trial Center, Soonchunhyang University College of Medicine, Cheonan Hospital, Cheonan-si, KR

**Keywords:** renal artery, embolization, therapeutic, acute kidney injury, renal insufficiency, chronic, hypertension

## Abstract

**Objectives:**

To evaluate the effect of superselective renal artery embolization in terms of renal function and blood pressure, to compare the results between groups with different embolization extents, and to analyze risk factors of entire study population for postprocedural acute kidney injury (AKI).

**Materials and Methods:**

The inclusion criteria were patients who underwent renal artery embolization from January 2009 to December 2019, with available serum creatinine and blood pressure data. The exclusion criteria were non-selective embolization of main renal artery, AKI before embolotherapy, and follow-up of less than one month. According to the extent of embolization, the patients were divided into two groups: Group A (1 segment) and Group B (2–4 segments).

**Results:**

A total of 48 patients were enrolled. There was a significant difference between pre- and postprocedural estimated glomerular filtration rate (*p* = 0.030). There were no significant difference between pre- and postprocedural blood pressure. The incidence of postprocedural AKI in group B was significantly higher than that in group A (*p* = 0.044). There was no significant difference in the incidence of the worsening of hypertension between the two groups. Chronic kidney disease and high embolization grade were predictive for postprocedural AKI (*p* = 0.012, 0.021).

**Conclusion:**

Superselective embolization appears to be a safe procedure, but meticulous attention for AKI is required for patients who underwent embolization of more than one segmental artery. An attempt to minimize the extent of devascularization should be pursued to avoid postprocedural complications.

## Introduction

Renal artery embolization (RAE) is a minimally invasive therapeutic option that can be used to treat a variety of conditions. A few previous studies have found RAE to be a safe procedure, but renal failure and hypertension (HTN) are possible alarming complications [1,2,3]. It is expected that advances in endovascular techniques could reduce the loss of nephrons and complications. However, superselective embolization is sometimes regarded as a time-consuming and unreasonable step in patients with difficult anatomy or unstable vital signs. The current single-institution study aimed to evaluate the effect of superselective RAE in terms of renal function and blood pressure, to compare clinical outcomes between groups with different embolization extents, and to investigate risk factors of the entire study population for postprocedural acute kidney injury (AKI).

## Materials and Methods

**Figure 1:**
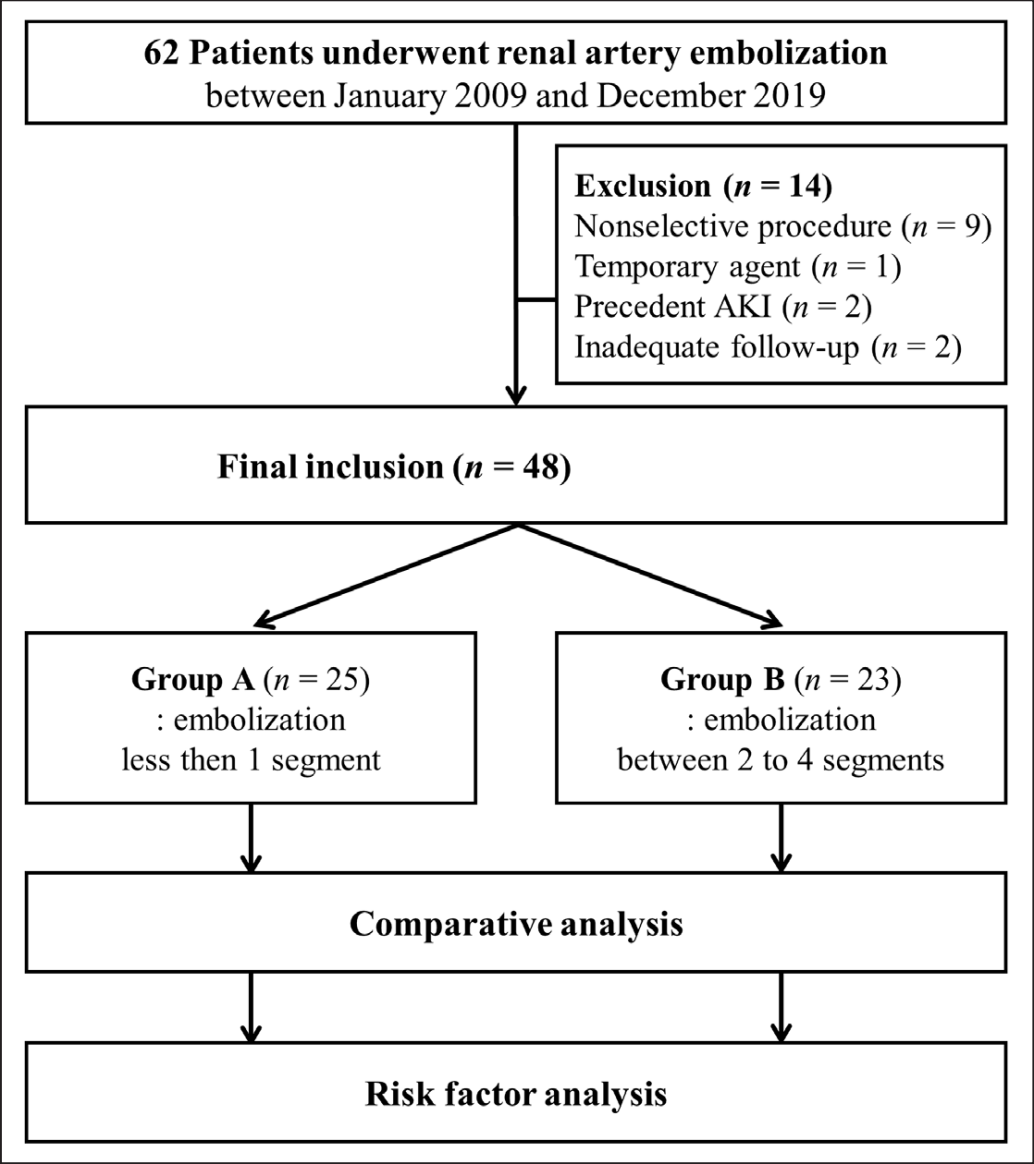
Study flow diagram.

The institutional review board at our hospital approved this retrospective single-center study and waived written informed consent for the use of clinical and imaging data. The flow diagram of the current study is depicted in Figure 1.

### Study Patients and Data Collection

**Figure 2:**
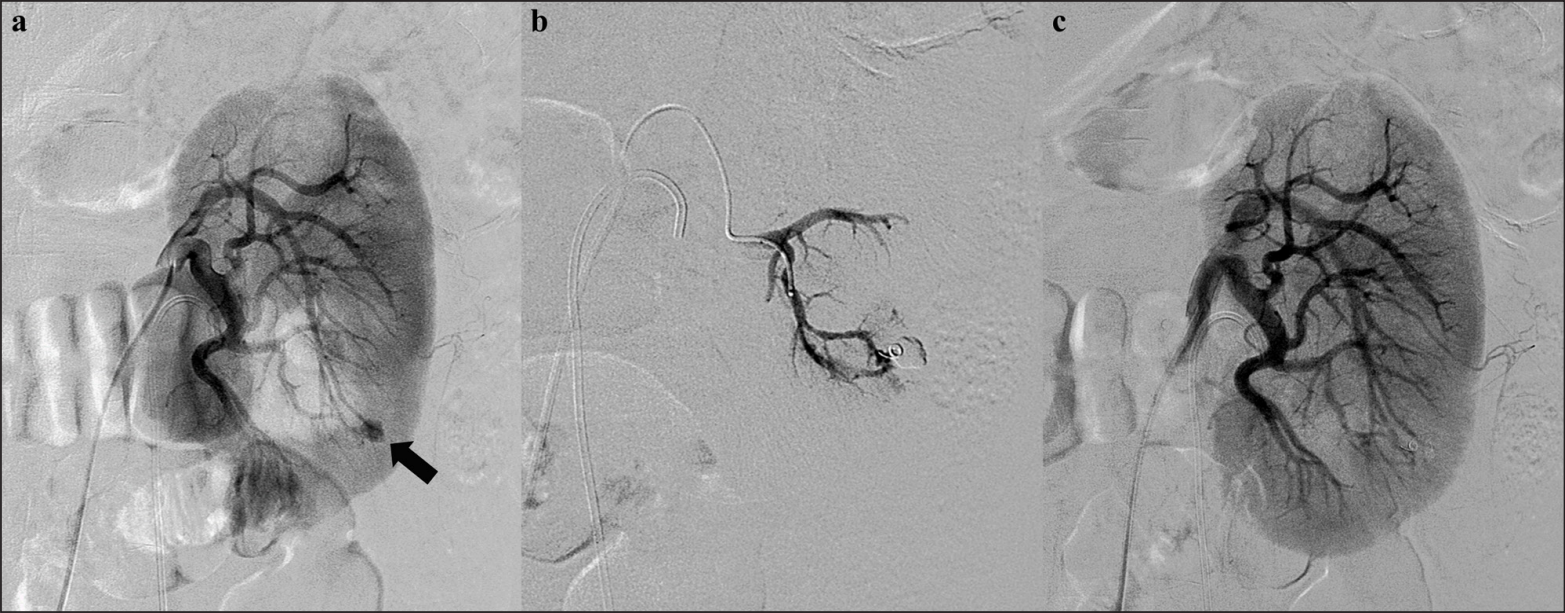
A 54-year-old man presenting with persistent hematuria after percutaneous nephrolithotomy. **(a)** An initial left renal angiogram shows a small pseudoaneurysm (black arrow) at arcuate branch of posterior segmental artery. **(b)** Superselective embolization of feeding artery using coaxial microcatheter system and a microcoil. **(c)** Postprocedural angiogram demonstrates complete devascularization of pseudoaneurysm.

**Figure 3:**
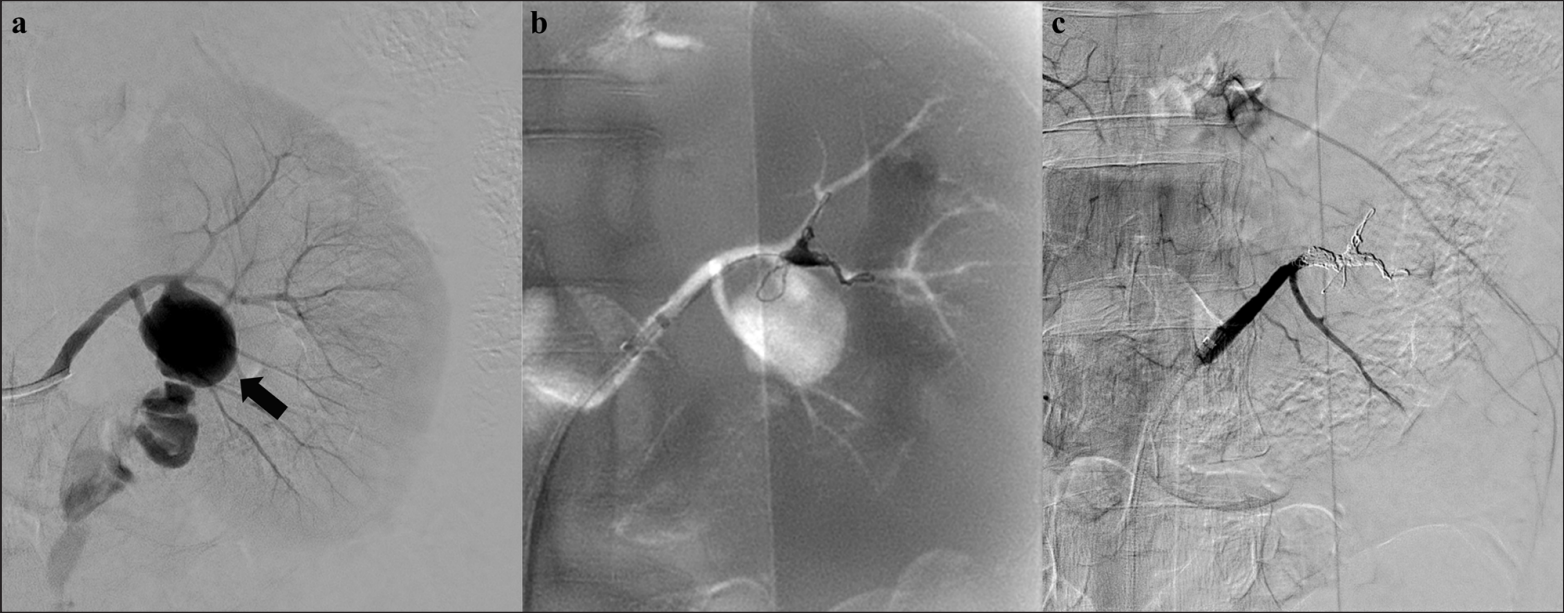
A 49-year-old woman presenting with acute left flank pain without trauma history. **(a)** An initial left renal angiogram reveals a huge pseudoaneurysm (black arrow) and contrast extravasation at proximal segment of anterior division. **(b)** Superselective embolization of anterior division using coaxial microcatheter system and detachable microcoils. **(c)** Complete cessation of active bleeding on final angiogram with preserved flow of posterior division.

The inclusion criteria were (i) all patients who underwent therapeutic RAE from January 2009 to December 2019, (ii) patients with documented pre- and postprocedural laboratory data of serum creatinine (S_Cr_) and (iii) patients with clinical blood pressure data. The exclusion criteria were (i) non-selective embolization of main renal artery, (ii) procedures using only temporary embolic agent, (iii) AKI before embolotherapy and (iv) follow-up period of less than one month. The patients were divided into two groups according to the grade of embolization extent: Grade 1 as group A and Grade 2 as group B. The extent of embolization was graded according to embolized vascular territory using a 2-point scale on angiography: Grade 1 (1 segmental artery, Figure 2) and Grade 2 (2–4 segmental arteries, Figure 3).

Electronic medical records of all patients were reviewed. The laboratory and clinical data were collected on pre- and postprocedural day 2, day 90, and the last follow-up. The estimated glomerular filtration rate (eGFR) was calculated using the Chronic Kidney Disease Epidemiology Collaboration equation as follows: (141 x min (S_Cr_/κ, 1)^α^ × max (S_Cr_/κ, 1)^–1.209^ × 0.993^Age^ × 1.018 [if female] × 1.159 [if Black], κ = 0.7 (female) or 0.9 (male)) [4]. The eGFR values are expressed as mL/min/1.73 m^2^. According to the guidelines of the Kidney Disease Improving Global Outcomes, AKI was defined as an increase in S_Cr_ value of ≥ 0.3 mg/dL (26.5 μmol/L) or ≥50% increase in S_Cr_ within 48 h [5]. Chronic kidney disease (CKD) was defined as eGFR < 60 mL/min/1.73 m^2^ for three months or more.

Maximum acceptable contrast dose (MACD) was defined as (5 mL × body weight (kg))/baseline S_Cr_ (mg/dL)) according to the study of Cigarroa et al. [6]. The ratios of contrast volume: MACD (CV/MACD) of each patient were calculated [7,8]. A ratio >1 indicated that the given contrast volume exceeded the MACD threshold.

According to the European Society of Cardiology/European Society of Hypertension guidelines, HTN was defined as two or more abnormal blood pressure readings ≥140/90 mmHg and/or antihypertensive management [9]. We defined the worsening of HTN as when mean arterial pressure increased ≥10 mm Hg or when additional antihypertensive medications were required after the procedure [1].

### Routine Procedural Technique

Renovascular anatomy and target lesions were carefully reviewed on preprocedural imaging. All procedures were performed by two experienced interventional radiologists. A 5-F vascular sheath was placed in the common femoral artery. Renal angiography was performed using a 5-F catheter, and angiographic findings were evaluated. Superselective embolization was performed on culprit arteries via a 2.0- to 3.0-F microcatheter with special attention to avoid inadvertent embolization. The contrast agent used was Visipaque (iodixanol, 320 mg/mL, Nycomed, Princeton, NJ) in all procedures.

### Statistics

For descriptive statistics, numerical variables are presented as the mean ± standard deviation and categorical variables are presented as absolute and relative frequencies. The Wilcoxon signed rank test or paired sample *t*-test was used to compare pre- and postprocedural values. Binary logistic regression was used to determine the risk factors for postprocedural AKI. A multivariable analysis model was built using a forward selection. We calculated the variance inflation factor (VIF). The diagnostic performance of the multivariable logistic regression model was assessed using the area under the curve (AUC) of receiver operating characteristic (ROC) curves. For comparative analysis between the two groups, the two-sample *t*-test or Mann-Whitney U test was used for continuous variables and the chi-square test or Fisher’s exact test for categorical variables. A *p*-value <0.05 was considered statistically significant. The statistical analyses were executed using R (version 3.6.0 software; Foundation for Statistical Computing, Vienna, Austria).

## Results

A total of 62 patients were reviewed, and 48 consecutive patients (29 men, overall mean age 48.4 years) met the inclusion criteria. According to the extent of embolization, the patients were divided into two groups: group A (*n* = 25) and group B (*n* = 23). There was no significant difference in baseline characteristics between the two groups (Table 1). Embolotherapy was technically successful in all patients. Indications for the procedure and embolic materials used are summarized in Table 2. The median follow-up period was 216 days (range, 1–109.5 months).

**Table 1:** Baseline characteristics of the whole study population.

Characteristics	Total	Group A	Group B	*p*-value

	(*N* = 48)	(*n* = 25)	(*n* = 23)	

Age	48.40 ± 17.14	49.04 ± 17.82	47.70 ± 16.74	0.789
Sex (male)	29 (60.4%)	15 (60%)	14 (60.9%)	>0.99
DM	9 (18.8%)	6 (24%)	3 (13%)	0.466
SBP	123.12 ± 17.03	123.20 ± 14.92	123.04 ± 19.41	0.545
DBP	74.79 ± 9.22	74.80 ± 8.23	74.78 ± 10.39	0.861
eGFR	81.84 ± 41.66	78.09 ± 42.81	85.92 ± 40.93	0.726
CV/MACD	0.37 ± 0.34	0.35 ± 0.33	0.40 ± 0.36	0.243

DM: diabetes mellitus, SBP: systolic blood pressure, DBP: diastolic blood pressure.

**Table 2:** Indications for the procedure and embolic materials used.

Indications	Number of procedures

Aneurysm	1
Angiomyolipoma	17
Arteriovenous malformation	2
Iatrogenic injury	17
Spontaneous kidney rupture	6
Traumatic injury	5
**Embolic material**	**Number of procedures**

Coil	21
Polyvinyl alcohol particles	15
N-butyl-2-cyanoacrylate	2
Multiple agents	10

### Renal function and blood pressure

Twelve patients (25%, 12/48) had underlying CKD. The baseline eGFR for the entire study group was 81.84 ± 41.66 mL/min/1.73 m^2^, and the eGFR at the last follow-up was 77.14 ± 36.90 mL/min/1.73 m^2^ (Table 3). The difference between pre- and postprocedural eGFR (–4.7 ± 17.57 mL/min/1.73 m^2^) was statistically significant (*p* = 0.030). Seven patients (14.6%, 7/48) experienced postprocedural AKI. Among these, five patients had underlying CKD. In all patients with postprocedural AKI, eGFR returned to baseline level within 6–13 postprocedural days (mean 10 days).

**Table 3:** Pre- and postprocedural eGFR and blood pressure.

	Total	Group A	Group B	*p*-value

	(*N* = 48)	(*n* = 25)	(*n* = 23)	

**Postprocedural AKI**				
Incidence	7 (14.6%)	1 (4%)	6 (26.1%)	**0.044**
**eGFR (mL/min/1.73 m^2^)**				
Baseline	81.84 ± 41.66	78.09 ± 42.81	85.92 ± 40.93	0.726
Postprocedure	77.14 ± 36.90	77.54 ± 39.52	76.70 ± 34.70	0.549
Mean difference	–4.70 ± 17.57	–0.55 ± 16.98	–9.22 ± 17.45	0.089
*p*-value	**0.030**	0.872	**0.019**	
**Postprocedural HTN**				
Incidence	3 (6.3%)	0 (0%)	3 (13%)	0.102
**SBP (mmHg)**				
Baseline	123.12 ± 17.03	123.20 ± 14.92	123.04 ± 19.41	0.545
Postprocedure	123.31 ± 16.44	122.84 ± 17.49	123.83 ± 15.60	0.837
Mean difference	0.19 ± 11.79	–0.36 ± 10.05	0.78 ± 13.64	0.751
*p*-value	0.913	0.661	0.786	
**DBP (mmHg)**				
Baseline	74.79 ± 9.22	74.80 ± 8.23	74.78 ± 10.39	0.861
Postprocedure	74.67 ± 11.18	74.92 ± 11.22	74.39 ± 11.39	0.915
Mean difference	–0.12 ± 10.92	0.12 ± 8.86	–0.39 ± 12.99	0.658
*p*-value	0.735	0.724	0.887	

SBP: systolic blood pressure, DBP: diastolic blood pressure.

Sixteen patients (33.3%, 16/48) had underlying HTN. The baseline systolic blood pressure (SBP) for the entire study group was 123.12 ± 17.03 mmHg, and the diastolic pressure (DBP) was 74.79 ± 9.22 mmHg. At the last follow-up, the SBP was 123.31 ± 16.44 mmHg, and the DBP was 74.67 ± 11.18 mmHg. The differences between pre- and postprocedural SBP (0.19 ± 11.79 mmHg, *p* = 0.913) and DBP (–0.12 ± 10.92 mmHg, *p* = 0.735) were not statistically significant. The worsening of HTN occurred in three patients (6.3%). Two patients required the initiation of antihypertensive therapy, and another patient required additional antihypertensive agents after the procedure.

### Subgroup analysis

Subgroup analysis revealed significant decrease in postprocedural eGFR in group B (–9.22 ± 17.45 mL/min/1.73 m^2^, *p* = 0.019). There was no significant change in postprocedural blood pressure in either group. On comparative analysis, the incidence of postprocedural AKI in group B (26.1%) was significantly higher than that in group A (4%, *p* = 0.044). There was no significant difference in the incidence of the worsening of HTN between the two groups.

### Risk factors for postprocedural AKI

**Figure 4:**
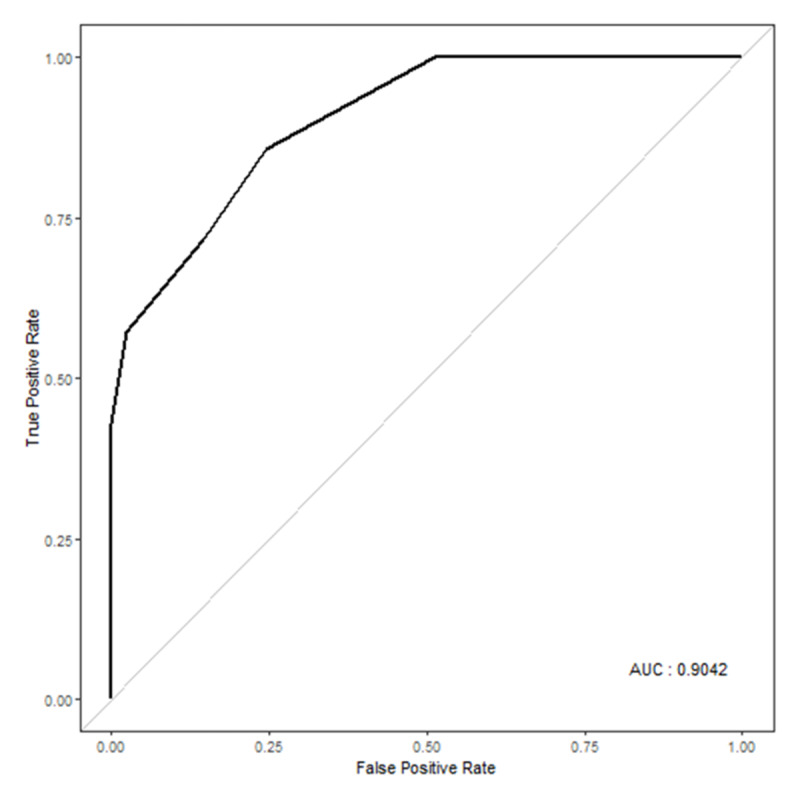
ROC curve for the multivariable logistic regression analysis.

In the multivariable binary logistic analysis of entire study population, CKD (OR: 35.67, 95% CI: 2.22–572.50, *p* = 0.012) and embolization grade 2 (OR: 48.53, 95% CI: 1.79–1318.57, *p* = 0.021) were predictive of postprocedural AKI (Table 4). There was no multicollinearity because the VIF was less than the threshold value of 10. The AUC indicated good performance (0.904), as in Figure 4.

**Table 4:** The logistic regression model for risk factors of AKI.

Variables	Univariable analysis			Multivariable analysis			

	OR	95% CI	*p*-value	OR	95% CI	*p*-value	VIF

Age	0.98	0.94–1.03	0.433				
Sex (male)	4.82	0.83–28.09	0.081	5.50	0.45–67.14	0.182	1.17
DM	4.38	0.78–24.66	0.094				
HTN	6.82	1.15–40.41	0.035				
CKD	12.14	1.95–75.73	0.008	35.67	2.22–572.50	**0.012**	1.58
Embolization extent	2.14	0.93–76.89	0.058	48.53	1.79–1318.57	**0.021**	1.75
CV/MACD	15.51	1.86–129.68	0.011				

## Discussion

A considerable number of patients undergoing RAE may have underlying kidney disease or HTN. The best available evidence is from a retrospective study by Collins et al., which demonstrated no significant change in renal function or blood pressure [1]. However, they included patients with precedent AKI and patients who underwent embolization with temporary embolic agent in the study group. In the current study, we excluded patients with aforementioned conditions to improve the homogeneity of the study population.

Our study demonstrated a significant postprocedural decrease in eGFR in the overall study group (–4.7 ± 17.57 mL/min/1.73 m^2^, *p* = 0.030) and in group B (–9.22 ± 17.45 mL/min/1.73 m^2^, *p* = 0.019). Although no patient experienced postprocedural CKD, superselective RAE appeared to cause a long-term decrease in eGFR, especially in patients with a large embolization extent. Comparative analysis also demonstrated this trend (*p* = 0.09). The discrepancy from the previous study [1] might be explained by 1) exclusion of patients with existing AKI at the time of study enrollment and 2) stratification according to embolization extent. Our study results are partly in accordance with counterpart studies in terms of surgical outcomes, which demonstrated a reduced incidence of CKD in patients who underwent nephron-sparing surgery [10].

AKI is one of the most serious complications after RAE. Patients who have experienced AKI are particularly vulnerable to poor long-term renal outcomes [11]. In the current study, embolization extent was identified as a modifiable risk factor for postprocedural AKI. We can assume that postprocedural AKI occurs almost exclusively in patients who underwent embolization of more than one segment. In the literature, it has been recognized that underlying CKD and a large volume of contrast media are major risk factors for postprocedural AKI [8,12]. Although CV/MACD was not significant on multivariable analysis, the half of patients (50%, 2/4) with high CV/MACD (>1) experienced postprocedural AKI in the current study. It would be best to limit the contrast dose to lower than the MACD; however, carbon dioxide angiography might be used as a useful alternative contrast agent in selected cases [13].

The pathogenesis of HTN following RAE is not clear, and the existing evidence appears to be limited to case reports and case series [14,15,16]. The kidneys play an integral role in the regulation of blood pressure and fluid balance. Hemodynamic alteration after embolization may result in a chronic hypertensive state [22]. Some have argued that incomplete renal infarction may stimulate the renin-angiotensin system of ischemic renal parenchyma [14]. On the other hand, our results are in line with the aforementioned study of RAE in that blood pressure was preserved after the procedure [1].

The current study has several limitations. Because of its retrospective nature, there could be bias in the data collection and analyses. Patients with various indications were enrolled in study group and the number of patients with each indication was too small for subgroup analyses. The follow-up period of each patient varied widely. The analysis of postprocedural CT findings can be more predictive for residual renal function. However, the detailed extent of embolization is determined according to the preprocedural angiographic finding.

## Conclusion

Superselective embolization appears to be a safe procedure, but meticulous attention for AKI is required for patients who underwent embolization of more than one segmental artery. An attempt to minimize the extent of devascularization should be pursued to avoid postprocedural complications.
